# Single-cell genomics unveils a canonical origin of the diverse mitochondrial genomes of euglenozoans

**DOI:** 10.1186/s12915-021-01035-y

**Published:** 2021-05-17

**Authors:** Kristína Záhonová, Gordon Lax, Savar D. Sinha, Guy Leonard, Thomas A. Richards, Julius Lukeš, Jeremy G. Wideman

**Affiliations:** 1 Institute of Parasitology, Biology Centre, Czech Academy of Sciences, České Budějovice (Budweis), Czech Republic; 2 Faculty of Science, Charles University, BIOCEV, Vestec, Czech Republic; 3 Department of Botany, University of British Columbia, Vancouver, Canada; 4 Center for Mechanisms of Evolution, Biodesign Institute, School of Life Sciences, Arizona State University, Tempe, USA; 5 Department of Zoology, University of Oxford, Oxford, UK; 6 Faculty of Sciences, University of South Bohemia, České Budějovice (Budweis), Czech Republic

**Keywords:** Single-cell amplified genome, Evolution, Mitochondrial ribosome, Phylogeny

## Abstract

**Background:**

The supergroup Euglenozoa unites heterotrophic flagellates from three major clades, kinetoplastids, diplonemids, and euglenids, each of which exhibits extremely divergent mitochondrial characteristics. Mitochondrial genomes (mtDNAs) of euglenids comprise multiple linear chromosomes carrying single genes, whereas mitochondrial chromosomes are circular non-catenated in diplonemids, but circular and catenated in kinetoplastids. In diplonemids and kinetoplastids, mitochondrial mRNAs require extensive and diverse editing and/or *trans*-splicing to produce mature transcripts. All known euglenozoan mtDNAs exhibit extremely short mitochondrial small (*rns*) and large (*rnl*) subunit rRNA genes, and absence of tRNA genes. How these features evolved from an ancestral bacteria-like circular mitochondrial genome remains unanswered.

**Results:**

We sequenced and assembled 20 euglenozoan single-cell amplified genomes (SAGs). In our phylogenetic and phylogenomic analyses, three SAGs were placed within kinetoplastids, 14 within diplonemids, one (EU2) within euglenids, and two SAGs with nearly identical small subunit rRNA gene (18S) sequences (EU17/18) branched as either a basal lineage of euglenids, or as a sister to all euglenozoans. Near-complete mitochondrial genomes were identified in EU2 and EU17/18. Surprisingly, both EU2 and EU17/18 mitochondrial contigs contained multiple genes and one tRNA gene. Furthermore, EU17/18 mtDNA possessed several features unique among euglenozoans including full-length *rns* and *rnl* genes, six mitoribosomal genes, and *nad11*, all likely on a single chromosome.

**Conclusions:**

Our data strongly suggest that EU17/18 is an early-branching euglenozoan with numerous ancestral mitochondrial features. Collectively these data contribute to untangling the early evolution of euglenozoan mitochondria.

**Supplementary Information:**

The online version contains supplementary material available at 10.1186/s12915-021-01035-y.

## Background

The major components of the eukaryotic tree of life that remain underexplored comprise heterotrophic flagellates (HFs) [1]. Investigation of HFs enables improved understanding of both eukaryotic evolution and diversity. However, traditional methods for acquiring molecular data from HFs necessitates the establishment of cultures, which can be difficult [1], especially as many of these cellular forms depend on symbiotic interactions. New approaches have focused on leveraging single-cell amplified genome (SAG) sequencing technologies to generate data enabling placement of these mysterious groups into the tree of life [2–4]. Single-cell and traditional approaches have recently uncovered HFs that are not part of well-studied groups (e.g., *Rhodelphis*, SAG D1, *Ancoracysta*) [2, 5, 6], and phylogenetic placement of orphan taxa has helped to resolve major branching relationships (e.g., Hemimastigophora, Picozoa, telonemids) [7–9]. Further sampling of HFs is predicted to shed light on key evolutionary transitions that led from ancestral to diverged states across the eukaryotes.

Among HFs, the phylum Euglenozoa includes lineages with some of the most divergent cellular characteristics. Euglenozoans comprise four well-defined clades: kinetoplastids, diplonemids, euglenids, and symbiontids [10–12]. Most euglenozoans, including diplonemids [13, 14], most kinetoplastids [15], and most euglenids are free-living HFs [16, 17]. Symbiontids are anaerobic and covered in epibiotic bacteria [10, 11]. Investigations of the parasitic kinetoplastid *Trypanosoma brucei* have shown that although their mitoribosomes, nuclear pore complexes, and mitochondrial import machineries are extremely diverged from most other eukaryotes, they function in a similar manner [18–20]. Increased sampling of rare euglenozoans that branch at the base of each major lineage will provide insights into how these cellular characteristics diversified in structure and protein composition, without obvious functional divergence.

Compared to lineages like vertebrates and fungi, in which relatively little mitochondrial genome (mtDNA) variation has occurred, euglenozoans have amassed mitochondrial genomic oddities like no other eukaryotic group [21]. Juxtaposed with their closest relatives (i.e., jakobids, *Tsukubamonas*, and heteroloboseans), which have bacteria-like, circular-mapping, gene-rich mtDNAs, the transcripts of which do not require *trans*-splicing or RNA editing [22–25], euglenozoans exhibit diverse mtDNA architectures and mitochondrial transcript processing mechanisms [26–29]. The mtDNA of the euglenid *Euglena gracilis* is composed of linear chromosomes bearing full-length genes, non-functional pseudogenes, and terminal repeats in diverse arrangements [28, 30]. Kinetoplastid mtDNA, like that of *T. brucei*, is split into mutually interlocked maxi- and minicircles [31]. Maxicircles contain unrecognizable mitochondrial genes, the transcripts of which undergo extensive uridine insertion/deletion editing, facilitated by minicircle-encoded guide RNAs and complex protein machinery [27]. Diplonemids have mitochondrial genes split into small fragments, often on separate molecules [32, 33], which require extensive *trans*-splicing and four kinds of RNA editing to produce mature transcripts [29, 32–34]. How this diversity evolved in euglenozoans is unclear.

A recently sequenced SAG (called “D1”) branched sister to all known kinetoplastids in phylogenetic analyses [2]. Its mitochondrial contigs contained genes that were fragmented in a similar fashion as diplonemids, requiring diplonemid-like *trans*-splicing to produce mature transcripts, but lacked any RNA editing [2]. While these data allow us to infer that the ancestor of kinetoplastids and diplonemids had a diplonemid-like mtDNA architecture, we cannot infer how the various idiosyncrasies of euglenozoan mtDNAs emerged from a canonical architecture. Here, we provide a starting point for understanding early transitions by identifying a deep-branching euglenozoan SAG with an ancestral-like mtDNA lacking any RNA editing and/or *trans*-splicing, which encodes full-length mitoribosomal genes, and multiple mitochondrial genes not present in other euglenozoan mtDNAs.

## Results and discussion

### Diverse euglenozoans can be recovered in SAG analyses

We sequenced and assembled 20 SAGs previously amplified and reported to be euglenozoans via V9 18S mapping [4]. Assemblies varied in size from 1.2 Mb (EU20) to 57.6 Mb (EU16) (Additional file 1: Table S1). Similar to previous reports [2, 35, 36], our SAGs had very low BUSCO completion scores (0–26% including fragmented genes) with the eukaryota_odb9 dataset (Additional file 1: Table S1). Although all SAGs contained predominantly eukaryotic sequences, six (EU3–5, EU7, EU15, and EU19) were judged to be heavily contaminated as they had relatively high (53–83%) BUSCO completion scores when using the bacteria_odb9 dataset. These results were consistent with the BlobTools plots (Additional file 2: Fig. S1). When the eukaryotic small subunit rDNA (18S) genes from all 20 SAGs were used as BLAST queries against the NCBI nucleotide database, their top BLAST hits were all euglenozoan 18S rDNAs (Additional file 1: Table S1).

### 18S rDNA and concatenated nuclear phylogenies reveal a deep-branching euglenozoan

**Fig. 1:**
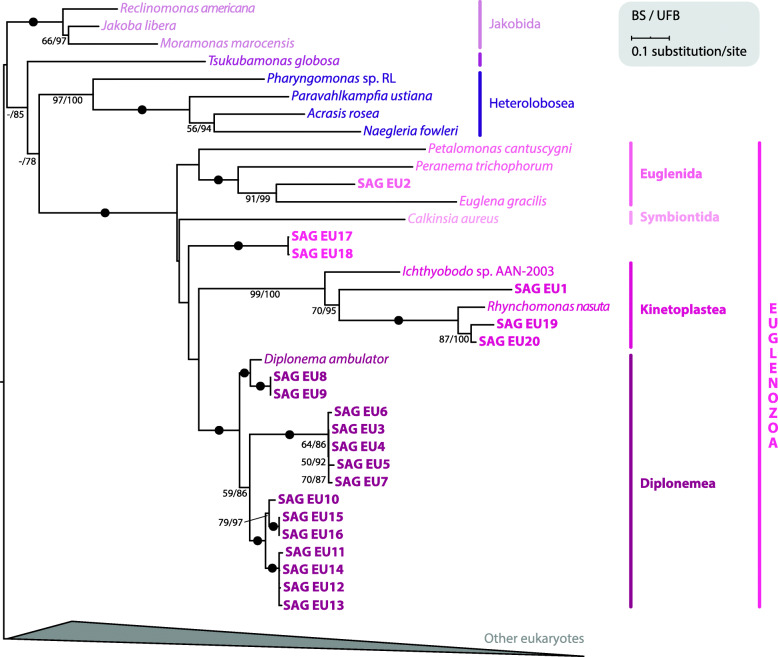
18S rDNA sequences from 20 SAGs branch with euglenozoans. 18S rDNA sequences were collected from 20 sequenced SAGs for phylogenetic analysis. The 18S rDNA alignment, based on a previously published dataset [7], contained 131 taxa and 1551 nucleotide positions. The maximum likelihood phylogenetic tree was estimated under GTR + Γ with standard bootstrapping (BS) and ultrafast bootstrapping (UFB). Support values are shown if ≥ 50% and ≥ 75% for BS and UFB, respectively. Fully supported nodes are shown as black circles. For clarity, the majority of eukaryotes are collapsed (for full tree see Additional file 2: Fig. S2)

Using a previously published 18S dataset [7], we performed phylogenetic analysis including 18S sequences from a broad diversity of eukaryotes (Fig. 1; Additional file 2: Fig. S2). All 20 SAGs branched within euglenozoans, which were recovered as a monophyletic clade with full support. The majority of these euglenozoan SAGs branched within major clades (3 kinetoplastids, 1 euglenid, and 14 diplonemids) with full support. Only SAGs EU17 and EU18 could not be confidently placed within any known euglenozoan clade. To better place these two SAGs, we conducted phylogenetic analyses with additional euglenozoan 18S (332 sequences) and 16 discobid sequences as an outgroup (Additional file 2: Fig. S3). Euglenozoan monophyly was obtained, and kinetoplastids, diplonemids, and symbiontids all formed monophyletic groups. As in some previous investigations [37, 38], euglenids were not retrieved as a monophyletic clade. EU2 branched sister to *Rapaza viridis*, confirming its euglenid identity (100% standard bootstrap [BS]). EU17 and EU18 branched with very low support (35% BS) sister to petalomonads, a basal euglenid lineage [39].

**Fig. 2:**
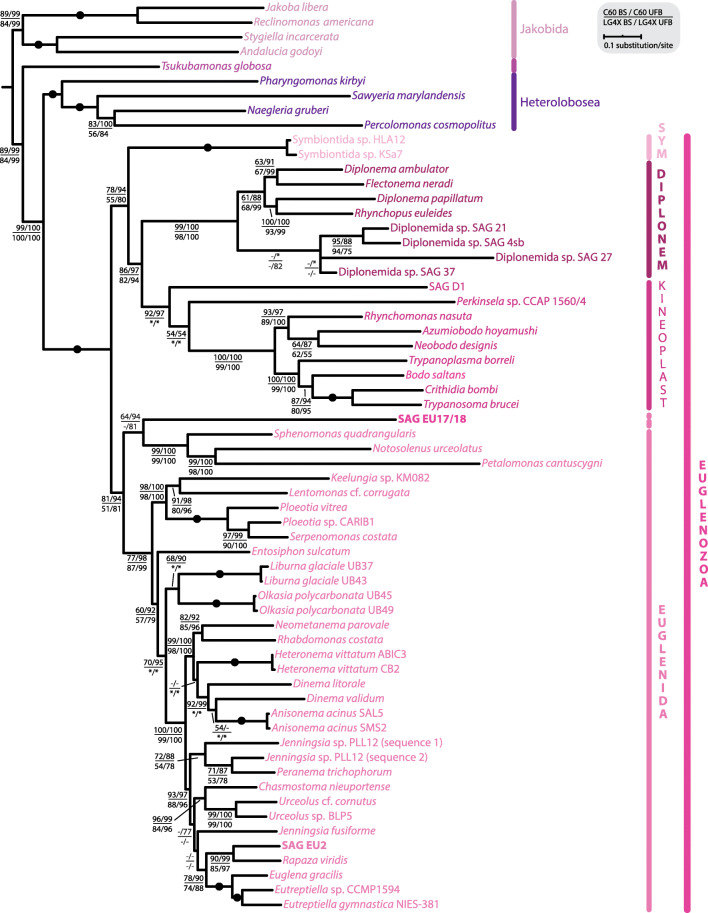
Concatenated nuclear phylogenetic analysis indicates EU17/18 represent a deep-branching euglenozoan. Assemblies of EU17/18 and EU2 were searched for a set of marker proteins based on a previously published study including 20 nucleus-encoded proteins [39] for phylogenetic analysis. The final alignment contained 62 taxa and 10,366 amino acid positions. EU17/18 and EU2 had 82% and 90% missing data, respectively. The maximum likelihood tree was estimated under two models, LG + C60 + F + Γ (C60) and LG4X, with standard bootstrapping (BS) and ultrafast bootstrapping (UFB). The tree topology shown is from the C60 analysis. Support values for < 50% BS and < 75% UFB are denoted by a dash (-), whereas an asterisk (*) marks a topology that does not exist in a particular analysis. Fully supported nodes are shown as black circles

To further investigate the position of EU17 and EU18 within euglenozoans, we performed a multi-gene phylogenetic analysis. Because their 18S sequences differed only in three nucleotides (not shown) and because of the low completeness of their individual assemblies (4.0% and 4.3% BUSCOs with eukaryota_odb9, respectively; Additional file 1: Table S1), we co-assembled EU17 and EU18 (Additional file 1: Table S1), producing SAG EU17/18 (6.9% eukaryota_odb9 BUSCOs). Of 20 nucleus-encoded proteins used in a recent euglenid study [39], only four and two of these genes were identified in EU17/18 and EU2, resulting in only 18% and 10% complete concatenated alignments, respectively. While EU2 branched again sister to *R. viridis* with 90% BS and 99% ultrafast bootstrap (UFB) support (LG + C60 + F + Γ), and 85/97% BS/UFB (LG4X), EU17/18 consistently formed a sister clade to petalomonads with moderate, but unconvincing support values given the extent of the missing data (64/94% BS/UFB using LG + C60 + F + Γ model, and 42/81% BS/UFB using LG4X model) (Fig. 2). Taken together, our results suggest that EU17 and EU18 likely represent a novel basal lineage of euglenids or a basal euglenozoan lineage. The uncertain placement could be attributed to the poor data representation, and/or the limited taxon sampling of basal euglenids and/or euglenozoans.

### Concatenated mitochondrial gene phylogeny places EU17/18 as a deep-branching euglenozoan

**Fig. 3:**
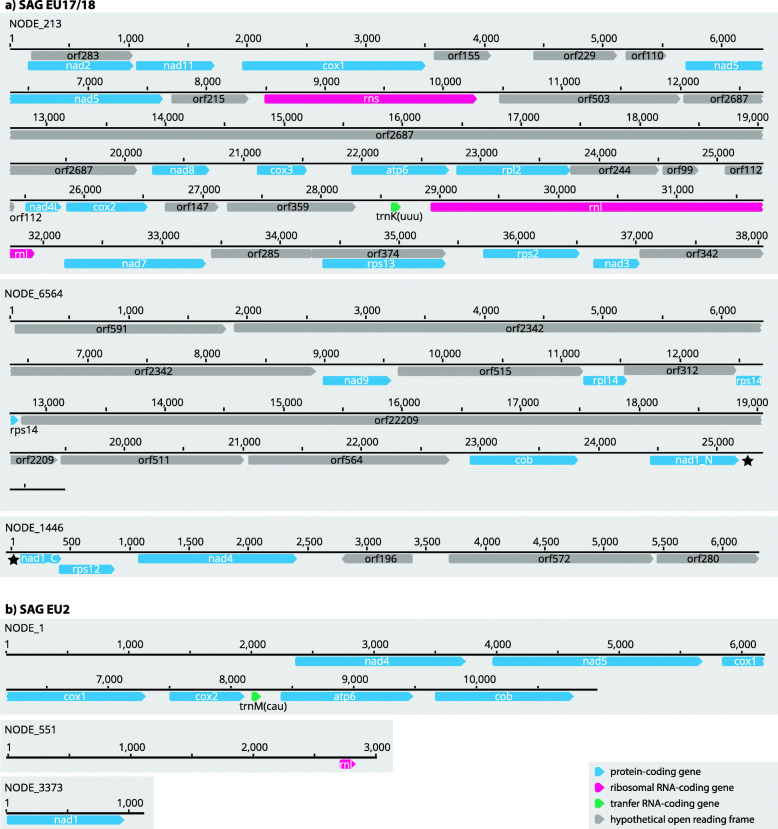
EU17/18 (**a**) and EU2 (**b**) mtDNA structure and gene content are unique among known euglenozoans. The reassembly of EU17/18 was obtained using the SAGAWE pipeline (see “Material and methods”) including reads from both EU17 and EU18 libraries. Horizontal lines represent mitochondrial chromosomes with genes as predicted by MFannot and lengths above them. Star (★) depicts the *nad1* gene that is split to two contigs most likely because of poor quality of contig ends

We identified 21 and seven ancestrally conserved mitochondria-encoded proteins in EU17/18 and EU2, respectively (Fig. 3). Full-length *rns* and *rnl* genes were identified in EU17/18 and a very small part of *rnl* (corresponding to the 3’ half [LSU-R] of the *E. gracilis rnl* [30]) was identified in EU2. Along with the standard mitochondrial genes, we identified a large number (24) of open reading frames (ORFs), that, when translated, have no similarity to any protein in the GenBank database. Furthermore, no protein domains were predicted by InterProScan, except transmembrane domains in 12 of them (Additional file 2: Fig. S4). One of these ORFs could represent an extremely divergent *nad6*, which is expected to be found as it is rarely lost or transferred to the nucleus [40]. No additional ORFs were identified in EU2 (Fig. 3). In both SAGs, several genes encoded TGA codons as tryptophan (W), a feature found in all known euglenozoans [28, 33, 41–43], further supporting the conclusion that the TGA-W genetic code change is an ancestral feature of euglenozoans. In addition, *trnK* was predicted to be encoded in EU17/18, and *trnM* in EU2 (Fig. 3).

**Fig. 4:**
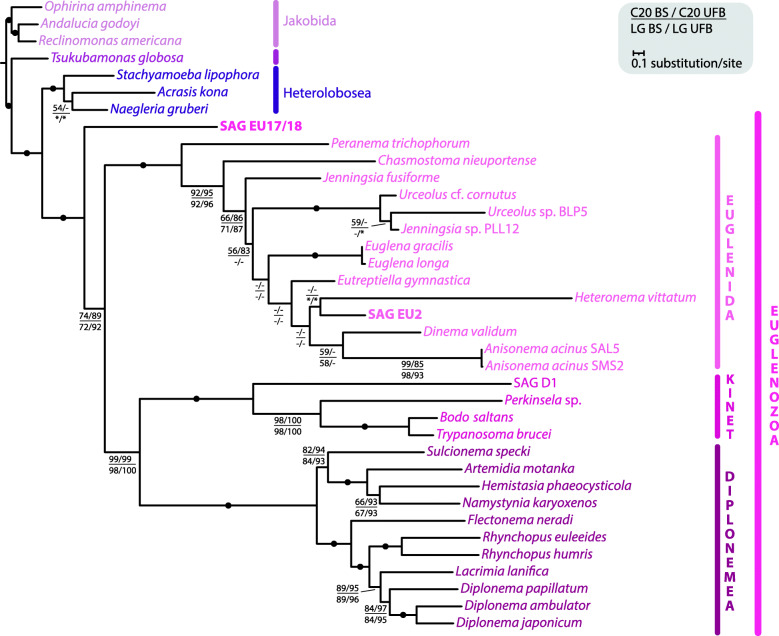
Concatenated mitochondrial phylogenetic analysis confirms EU17/18 as a member of an early-diverging euglenozoan lineage. Mitochondrial-encoded protein sequences were predicted from EU17/18 and EU2 mitochondrial contigs and subjected to phylogenetic analysis. The alignment, based on a previously published study including 15 mitochondrion-encoded proteins [33], contained 37 taxa and 4325 amino acid positions. EU17/18 and EU2 had 8.35% and 38.81% missing data, respectively. The maximum likelihood tree, estimated under two models, LG + C20 + F + Γ (C20) and LG + F + I + G4 (LG; the best-fitting model as determined by IQ-TREE), with 1000 standard bootstraps (BS) and 1000 ultrafast bootstraps (UFB). The tree topology shown is from the C20 analysis. Support values for < 50% BS and < 75% UFB are denoted by a dash (-), whereas an asterisk (*) marks a topology that does not exist in a particular analysis. Fully supported nodes are shown as black circles

Since the position of EU17/18 in nuclear phylogenies was not fully resolved, we sought to reconstruct a mitochondrial phylogeny using a set of 15 proteins used previously [33]. In this concatenated alignment, EU17/18 had only 8.35% missing data, whereas in EU2 38.81% was missing (in comparison, the model species *E. gracilis* had 32.42% missing data). The mitochondrial phylogeny shows euglenozoans, inclusive of EU17/18, as a fully supported monophyletic clade with fully supported euglenid (EU2 branching deeply within), kinetoplastid, and diplonemid clades (Fig. 4). Glycomonada (kinetoplastids + diplonemids) were also retrieved as monophyletic with nearly full support (99/99% BS/UFB using LG + C20 + F + Γ model, and 98/100% BS/UFB using LG + F + I + G4 model as determined by IQ-Tree as best-fitting model). Support for a monophyletic euglenozoan clade excluding EU17/18 was moderately supported (74/89% BS/UFB [LG + C20 + F + Γ], 72/92% BS/UFB [LG + F + I + G4]). These data further suggest that EU17 and EU18 represent an early-branching euglenozoan lineage. We repeated the analysis including the highly divergent *atp6* from EU17/18 identified using Phyre2. This phylogeny placed EU17/18 as a basal euglenid (Additional file 2: Fig. S5). However, the support for this position was low (72/85% [LG + C20 + F + Γ], 52/65% BS/UFB [LG + F + I + G4]), and many internal euglenid branches remained unresolved. While this placement is suggestive of a euglenid identity of EU17/18, the extreme divergence of *atp6* sequences in euglenozoans could also contribute to long-branch attraction artefacts. More mitochondrial genomes from deep-branching euglenids like petalomonads and ploeotids are required to improve the resolution of the deep-branching relationships within euglenozoans.

### EU17/18 and EU2 bear surprising mtDNA architecture and gene content

Since architectures of mitochondrial genomes differ significantly among euglenozoan lineages [26], we investigated the mtDNA contigs from EU2 and EU17/18 assemblies. Unlike other discobans, mitochondrial genome structures in euglenozoans exhibit diverse organizations [22]. The ancestral state from which these unique organizations evolved was likely a circular, gene-rich, bacteria-like mitochondrial genome. To date, no intermediates between the divergent extant euglenozoan architectures and their ancestral state have been discovered. Both EU2 and EU17/18 exhibited contigs encoding several mitochondrial genes, unscrambled, and without need for RNA editing (Fig. 3). To confirm these gene arrangements, we inspected read coverage by mapping trimmed reads onto contigs (see “Materials and methods”). Like previously sequenced SAGs [4], the mitochondrial to nuclear gene coverage ratios were very high in our euglenozoan SAGs (Additional file 1: Table S2), which is probably due to a combination of high mtDNA copy numbers in euglenozoans [44] as well as biassed amplification during SAG preparation. Nevertheless, read mapping showed a similar level of coverage throughout all contigs, supporting the predicted genome arrangements (Fig. 3).

All euglenozoans encode several subunits of respiratory complexes in their mitochondrial genome. Euglenid mtDNAs (e.g., *E. gracilis* and *Eutreptiella gymnastica*) encode only eight proteins: an extremely divergent ATP synthase subunit 6 (*atp6*), cytochrome *b* (*cob*), three subunits of cytochrome *c* oxidase (*cox1*, *cox2*, and *cox3*), and three subunits of NADH dehydrogenase (*nad1*, *nad4*, and *nad5*) [28, 45]. Diplonemids and kinetoplastids additionally encode seven subunits of NADH dehydrogenase (*nad2*, *nad3*, *nad4L*, *nad6*, *nad7*, *nad8*, and *nad9*) and kinetoplastids also possess two mitoribosomal proteins (*rps3* and *rps12*) as well as a protein of unknown function (*murf2*) [19, 46]. Diplonemids further encode two proteins with unknown function (*y4* and *y7*), although the former might represent a highly divergent subunit of the respiratory chain complexes or a mitoribosomal protein [32, 33, 47]. So far, no euglenozoan mtDNA was found to encode a tRNA gene [26, 48]. Small and large subunits of mitoribosomal RNAs (*rns* and *rnl*) of kinetoplastids are extremely short [49–51]. In euglenids, *rns* and *rnl* are each split into two halves that are separately transcribed, though they appear to fold into more conventional secondary structures [30]. Those in diplonemids are composed of one or more fragments, called modules (up to three and four for *rns* and *rnl*, respectively, depending on species) [32, 33]. Regardless of the number of modules, diplonemids’ *rns* are about 390 nt long, which is only one third of the length of those in kinetoplastids.

In the EU2 mitochondrial contigs, we were able to identify the identical protein-coding repertoire of genes as *E. gracilis* mtDNA with one exception (*cox3*) (Fig. 3). Several genes were identified on a single contig, namely *atp6*, *cob*, *cox1*, *cox2*, *nad4*, *nad5*, and surprisingly, a predicted tRNA^Met^(CAU) gene (Fig. 3; Additional file 2: Fig. S6). *nad1* and an *rnl* fragment were found on two separate contigs. Since EU2 is undoubtedly a euglenid (Figs. 1, 2, and 4; Additional file 2: Fig. S3), its gene arrangement suggests that the mitochondrial genome architecture in euglenids is not well reflected in the sole investigated species, *E. gracilis* [28, 30].

In EU17/18, we identified *atp6*, *cob*, all three *cox* subunits, 10 *nad* subunits (namely *nad1*, *nad2*, *nad3*, *nad4*, *nad4L*, *nad5*, *nad7*, *nad8*, *nad9*, and *nad11*), six mitoribosomal proteins (*rpl2*, *rpl14*, *rps2*, *rps12*, *rps13*, and *rps14*), full-length *rnl* and *rns*, and a single tRNA^Lys^(UUU) gene (*trnK*; Fig. 3; Additional file 2: Fig. S6). We were unable to identify *nad6*, which may have either diverged beyond recognition (e.g., *atp6* in *E. gracilis* [45]) or have independently transferred to the nucleus [52–57]. Unlike any other sequenced euglenozoan, EU17/18 exhibited full-length *rnl* and *rns* genes that could be easily aligned with ribosomal sequences of *E. coli* (Additional file 2: Fig. S7).

The gene repertoire and genome architecture of EU17/18 implies that the ancestral euglenozoan mtDNA was more complex than anticipated—in line with numerous convergent simplifications of mitochondrial gene content across the eukaryotes [6]. Our phylogenetic reconstructions suggest that EU17/18 represents either a deep-branching euglenozoan or a basal member of the euglenids. Either way, the euglenozoan ancestor can be inferred to have had a circular-mapping single mitochondrial chromosome encoding at least 16 electron transport chain components, seven mitoribosomal proteins (i.e., *rpl2*, *rpl14*, *rps2*, *rps3*, *rps12*, *rps13*, and *rps14*), full-length rRNA genes, and at least one tRNA.

### Euglenozoans exhibit a complex history of endosymbiont gene transfer of mitoribosomal proteins

Although genes encoding components of the electron transport chain and mitoribosomal proteins are retained in the mtDNA of most eukaryotes, these genes can be lost or transferred to the nucleus [58]. In sampled euglenozoans other than EU17/18, most protein components of mitoribosomes are encoded in the nuclear genome [19]. The only two exceptions are *rps3* and *rps12* genes in mtDNA of kinetoplastids, which could be retained in diplonemids but are too divergent to detect [19, 33]. In the well-characterized mitoribosome of the kinetoplastid *T. brucei*, the short length of mito-rRNAs is supplemented by 127 mitoribosomal proteins, assembled by a number of dedicated factors, making it the largest known ribosome [19, 59, 60]. While the *T. brucei* mitoribosome contains some conserved subunits widely distributed in other eukaryotes, it also incorporates numerous euglenozoa- and kinetoplastid-specific subunits [60–62]. The same applies for the *E. gracilis* mitoribosome, predicted to be composed of 108 proteins, also with euglenozoa- and euglenid-specific subunits present [63]. Indeed, there are eight eukaryotic mitoribosomal subunits in *E. gracilis*, which are absent from *T. brucei* (L1, L6, L18, L38, L56, S7, S13, and S28), and 16 eukaryotic subunits retained in *T. brucei*, but likely lacking in *E. gracilis* (L5, L29, L30, L33, L35, L41, L42, L48, S14, S21, S26, S30, S33, S37, S38, and Fyv4) [63, 64]. We sought to identify mitoribosomal genes encoded in the nuclear genome of EU17/18, but identified only one candidate *rps* protein (EG_transcript_13898 homologue) and two candidate *rpl* proteins (EG_transcript_7639 and EG_transcript_26116 homologues) (Additional file 1: Table S3). However, even if these proteins are orthologous, there is no guarantee that they will perform the same function as those in other euglenozoans. From where novel euglenozoan mitoribosomal proteins emerged and diversified is still unknown, but EU17/18 has provided a starting point for this investigation. As more sequences from the EU17/18 lineage become available, we will begin to grasp the origins of the divergent cellular features exhibited by euglenozoans.

## Conclusions

**Fig. 5:**
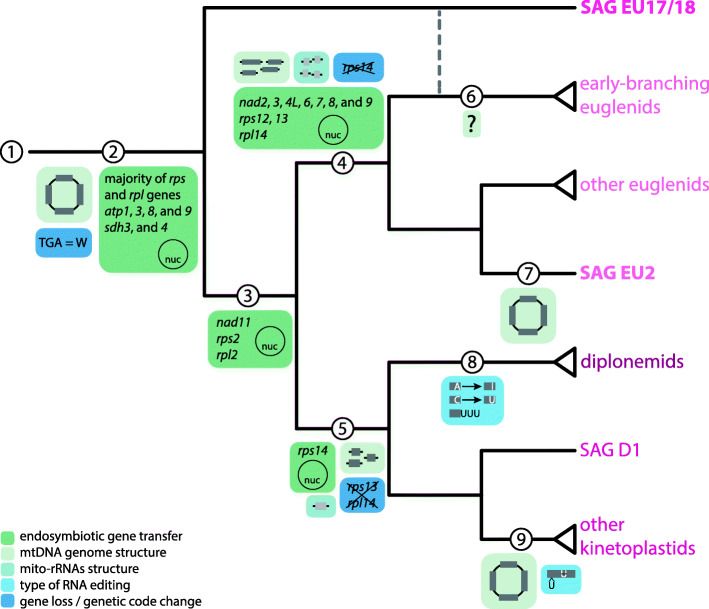
The evolutionary history of mtDNA in euglenozoans. Mitochondrial traits were mapped onto a schematic phylogenetic tree based on this and previous studies [2, 28, 30, 32, 43]. ① The ancestral discobid-like mtDNA. ② For the last common ancestor of euglenozoans, mtDNA was represented by a circular-mapping chromosome, meaning of TGA codon was changed to code for W, and genes for *atp1*, *3*, *8*, and *9*, *sdh3*, and *4*, and majority of mitoribosomal proteins were transferred to the nucleus. ③ After separation of EU17/18, *rps2*, *rpl2*, and *nad11* underwent endosymbiotic gene transfer (EGT). ④ For the common euglenid ancestor, mtDNA was fragmented to chromosomes with single genes and mito-rRNAs were split to halves. Majority of genes encoding NADH dehydrogenase, *rps12*, and *13*, and *rpl14* were transferred to the nucleus, and *rps14* was lost. ⑤ For the glycomonads, genes were split to modules and rRNAs were significantly shortened. While *rps14* underwent EGT, *rps13* and *rpl14* were lost completely. ⑥ Mitochondrial genome structure of early-branching euglenids is currently unknown. ⑦ For the SAG EU2, chromosomes encoding single genes were joined to a possibly circular molecule, resembling the ancestral state. ⑧ Diplonemids acquired diverse RNA editing mechanism to produce mature transcripts. ⑨ After separation of SAG D1, mtDNA of the rest of kinetoplastids was catenated into interlocked mini- and maxicircles. Primary transcripts are processed by U-insertion/deletion RNA editing to restore the coding sequence

Our data strongly suggest that SAG EU17/18 represents the earliest-branching lineage of known euglenozoans, or perhaps a novel euglenid lineage with ancestral features. EU17/18 exhibits several unique mitochondrial characteristics, enabling us to infer the ancestral euglenozoan mtDNA coding content and reconstruct the transformations that occurred within each major euglenozoan lineage (Fig. 5). From an ancestral heterolobosean-like mtDNA with a standard genetic code, several major changes can be traced (Fig. 5 ①). Prior to the last common euglenozoan ancestor the TGA-W genetic code change in mtDNA occurred and several genes were lost or transferred to the nucleus (②). The ancestral euglenozoan mtDNA was most probably a circular-mapping chromosome encoding *atp6*, *cob*, *cox1-3*, *nad1*, *2*, *3*, *4*, *4 L*, *5*, *6*, *7*, *8*, *9*, and *11*, *rpl2*, *14*, *rps2, 3*, *12*, *13*, *14*, full-length *rns* and *rnl*, as well as some tRNA genes (②). When tRNA genes were fully transferred to the nucleus remains unclear as the euglenid EU2 retains at least one predicted tRNA.

After divergence of EU17/18 from all other euglenozoans, *rps2*, *rpl2,* and *nad11* were transferred to the nucleus (③). From this point, major changes likely occurred in the *rns* and *rnl* genes, which coincided with the emergence of a number of novel mitoribosomal proteins [19, 59, 63]. In euglenids this can be seen by split *rns* and *rnl* genes ④, whereas in glycomonads, this is seen in their accelerated divergence and drastic shortening ⑤. In euglenids, *nad2*, *3*, *4 L*, *6*, *7*, *8*, and *9* were either lost or transferred to the nucleus, as only *atp6*, *cob*, *cox1*, *cox2*, *cox3*, *nad1*, *nad4*, and *nad5* were retained in the mtDNA ④ (more euglenid mtDNA sequences are needed to confirm this inference) [63]. In kinetoplastids and diplonemids, the mitochondrial coding complement is nearly overlapping in electron transport chain components, both retaining *atp6*, *cob*, *cox1*-*3*, *nad1*, *2*, *3*, *4*, *4 L, 5, 6, 7*, *8*, and *9* ⑤. Kinetoplastids additionally retain *rps3* and *rps12* [19, 46]. *rps13* and *rpl14* are encoded in the mtDNA of EU17/18, appear to be lost outright in glycomonads ⑤ but transferred to the nucleus in euglenids ④. Similarly, *rps14* was lost in euglenids ④ but likely transferred to the nucleus in the lineage leading to glycomonads ⑤.

**Fig. 6:**
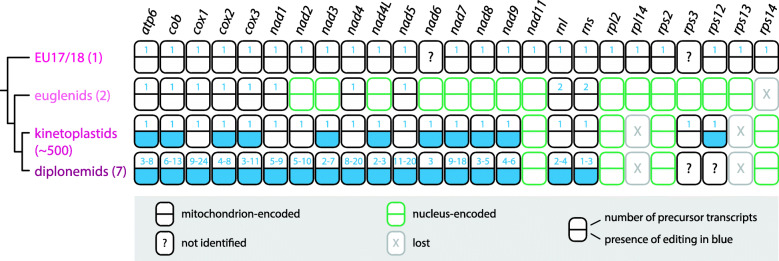
The mitochondrial genome of EU17/18 encodes the most complete mitochondrial repertoire of any known euglenozoan. Mitochondrion- and nucleus-encoded genes are shown as black and grey rectangles, as explained in graphical legend. For each gene, number of precursor transcript is indicated by number in the upper half of the rectangle. In case of diplonemids, the range indicates representatives with the smallest and highest number of modules as identified previously [32, 33]. Presence of any type of RNA editing is shown in blue in the lower half of the rectangle. The number in parentheses after the lineage name represents number of sequenced species

In conclusion, SAG EU17/18 has provided significant insight into the origin and diversification of the euglenozoan mtDNA gene complement (Fig. 6). The evolutionary reconstruction presented herein shows that the extreme complexity of mtDNA organization and transcript processing exhibited by most euglenozoans had rather simple origins, an unexpected finding for this group known for its highly divergent mitochondrial characteristics. Our data further demonstrate that single-cell approaches are necessary for identifying basal protist lineages whose incorporation into the tree of life is essential for understanding cellular evolution in particular and eukaryote biology in general.

## Material and methods

### Sample origin, genome amplification, and sequencing

We chose to sequence a set of 20 heterotrophic flagellate SAGs that were previously isolated and amplified, and their V9 18S sequences were Sanger sequenced [4]. V9 18S placement revealed their phylogenetic affinity to euglenozoans [4]. These SAGs originated from cells isolated from the deep chlorophyl maximum in the North Pacific Ocean as reported previously [4]. In the current study, we prepared sequencing libraries with Nextflex Rapid DNA library preparation kit (BIOO Scientific) and 12 cycles of PCR amplification. Libraries (250 bp paired-end) were sequenced on a HiSeq 2500 using Rapid run SBS v2 reagents (Illumina).

### Single-cell genome assembly

Each sequence library corresponding to a SAG was assembled using the SAGAWE pipeline available at https://github.com/guyleonard/sagawe. Briefly, libraries were quality controlled and adapter trimmed using Trim Galore! v0.6.4 (https://www.bioinformatics.babraham.ac.uk/projects/trim_galore), digitally normalised using BBNorm (part of the BBTools suite v37.93; https://jgi.doe.gov/data-and-tools/bbtools/) to compensate for uneven coverage of multiple displacement amplification, reads merged using BBMerge (part of the BBTools suite) to create sets of longer reads, and then assembled with SPAdes v3.13.1 [65] in single-cell mode under default parameters. Post assembly statistics were computed to provide general assembly stats using QUAST v5.0.2 [66], genome completeness estimates using BUSCO v3.0.1 [67] software, contamination checked using BlobTools v1.0 [68] and BLAST v2.2.31+ [69] against NCBI nucleotide database, and coverage estimated via read mapping using BWA v0.7.17-r1188 [70] and Qualimap v2.2.2-dev [71]. For the reassembly of EU17/18, the same pipeline was employed using reads from both EU17 and EU18 libraries.

### Phylogenetic analysis of 18S rDNA and concatenated nuclear genes

Small subunit ribosomal DNA (18S rDNA) was extracted from each assembly by BLASTn searches using a set of euglenozoan 18S sequences as queries. Extracted 18S rDNAs of SAGs were aligned using MAFFT v7.458 [72] under FFT-NS-i strategy (as determined with --auto) with 18S rDNAs of organisms from all major eukaryotic groups [7] and from other discobans. To remove poorly aligned positions trimAl v1.4 [73] (-gt 0.8) was used. Maximum likelihood analysis was performed with IQ-TREE v1.6.8 [74] under the GTR + Γ model. Branch supports were obtained by non-parametric (BS) and ultrafast (UFB) bootstrap approximation methods [75] with 100 and 1000 replicates, respectively.

Assemblies EU17/18 and SAG EU2 were queried for 20 nucleus-encoded proteins [39]. Found proteins were aligned in the respective single-gene alignment using MAFFT v7.458 [72] under L-INS-I strategy (as determined with --auto), and trimmed with trimAl v1.4 [73] (-gt 0.5). Single-gene trees were inferred from trimmed alignments using the best-fitting substitution model as determined by IQ-TREE v1.6.8 [74], and support assessed with 1000 ultrafast bootstrap replicates [75]. Single-gene trees were visually inspected to identify contaminant and paralogous sequences. The final trimmed and concatenated dataset consisted of 20 genes from 62 discobans and had 10,366 amino acid positions. A maximum likelihood phylogeny was estimated in IQ-TREE v1.6.8 [74] under two models: LG + C60 + F + Γ and LG4X. For the LG + C60 + F + Γ model, 200 BS and 1000 UFB replicates were used, and for the LG4X model, 1000 replicates were estimated with both bootstrapping methods.

### Mitochondrial genome analysis and concatenated phylogenetic analysis

Reassembly EU17/18 and SAG EU2 were searched for mitochondrial protein- and rRNA-coding genes using *Diplonema papillatum* [32] and *Euglena gracilis* [28] sequences as tBLASTn and BLASTn queries, respectively. The identity of found sequences was confirmed by BLAST searches against the NCBI non-redundant database. Larger mitochondrial contigs of EU2 and EU17/18 were analysed by MFannot (https://megasun.bch.umontreal.ca/cgi-bin/dev_mfa/mfannotInterface.pl). Protein domains were predicted by InterProScan search [76]. The protein translations of identified ORFs were subjected to search using Phyre2 [77] to identify more divergent homologues.

For presentation purposes, tRNA and rRNA sequences were aligned using the built-in aligner of the Geneious Prime v2020.2.3 software [78] and adjusted in a graphical editor.

Identified mitochondrial protein-coding genes were added to a dataset from a previous investigation [33]. Datasets were aligned with MAFFT v7.458 [72] under L-INS-I strategy (as determined with --auto), and trimmed with trimAl v1.4 [73] (-gt 0.5). Single-gene trees were inferred from the alignments using the best-fitting substitution model as determined by IQ-TREE v1.6.8 [74] and 1000 UFB replicates. The final trimmed concatenated dataset consisted of 15 genes from 37 discobans and had 4324 and 4348 amino acid positions with and without *atp6* from EU17/18, respectively. A maximum likelihood tree was inferred with IQ-TREE v.1.6.8 [74] under the LG + F + I + G4 model (determined as the best-fitting model by IQ-TREE) and 1000 BS and 1000 UFB replicates.

### Homology searching

Ancestral and euglenozoan-specific subunits of the mitoribosome were searched using the previously published dataset of *E. gracilis* [63]. Candidate homologues were subjected to a reciprocal BLAST search to validate orthology. A list of proteins that were searched is available in Additional file 1: Table S3.

## Supplementary Information

**Additional file 1: Table S1.** General statistics of assembled SAGs. **Table S2.** Proportion of mtDNA in SAGs. **Table S3.** List of proteins searched in EU17/18 reassembly. +, presence; +?, uncertain presence; -, absence.

**Additional file 2: Fig. S1.** BlobTools plots showing contamination of several sequenced SAGs. The bacterial contamination is shown as blue circles, while sequences with eukaryotic signal are in magenta. For comparison, BlobTools plots for SAGs EU17 and EU18 are also shown. **Fig. S2.** 18S rDNA phylogeny of eukaryotes. The Maximum Likelihood phylogenetic tree was estimated from an alignment containing 131 taxa and 1551 nucleotide positions under the GTR + Γ model with standard bootstrapping (BS) and ultrafast bootstrapping (UFB). Support values are shown if ≥ 50% and ≥ 75% for BS and UFB, respectively. Fully supported nodes are shown as black circles. **Fig. S3.** 18S rDNA phylogeny of euglenozoans. The Maximum Likelihood phylogenetic tree was estimated from an alignment containing 368 taxa and 1269 nucleotide positions under the GTR + Γ model with standard bootstrapping. Support values are shown if ≥ 50%. **Fig. S4.** Predicted domains in EU17/18 mtDNA-encoded ORFs. ORFs annotated by MFannot (https://megasun.bch.umontreal.ca/cgi-bin/dev_mfa/mfannotInterface.pl) were submitted to an InterProScan [76] search. Predicted domains are highlighted as explained in the graphical legend. **Fig. S5.** Concatenated mitochondrial phylogenetic analysis including *atp6* from EU17/18. The alignment contained 37 taxa and 4348 amino acid positions, with EU17/18 missing 3.65% of data. The Maximum Likelihood tree was estimated under two models, LG + C20 + F + Γ (C20) and LG + F + I + G4 (LG; the best-fitting model as determined by IQ-TREE), with 1000 standard bootstraps (BS) and 1000 ultrafast bootstraps (UFB). The tree topology shown is from the C20 analysis. Support values for < 50% BS and < 75% UFB are denoted by a dash (-), whereas an asterisk (*) marks a topology that does not exist in a particular analysis. Fully supported nodes are shown as black circles. **Fig. S6.** Characterization of tRNAs encoded in mtDNA of EU17/18 and EU2. a-b) Sequences of *trnK* (a) and *trnM* (b) were aligned with mitochondrially encoded tRNAs of other species of Discoba. Residue shading indicates sequence conservation. c-d) Secondary structures of *trnK* (c) and *trnM* (d) as predicted by tRNAScan-SE. Double and triple bonds are depicted as dark- and light-blue circles, respectively. Anticodons are highlighted with a green background. Since all other known euglenozoans import all tRNAs into mitochondria from the nucleus [48], we built tRNA alignments with homologues from the mtDNAs of other discobans to take into account different evolutionary pressures and mutational rates in nuclei and mitochondria [52]. The identity across nine *trnK* and 20 *trnM* sequences was 38.7% and 15.8%, respectively (a-b). Predicted secondary structures resembled other tRNAs supporting their functionality (c-d). While most eukaryotes have at least some tRNA mitochondrial-encoded, the long-standing paradigm was that euglenozoans and unrelated apicomplexans (which share with euglenozoans a range of unique features [79]) import all tRNAs from the cytosol [80]. This has significant consequences, since the bacterial-type translation system has to cope solely with the eukaryotic-type tRNAs [81]. **Fig. S7.** Mitoribosomal RNAs of EU17/18. Sequences of *rns* (a) and *rnl* (b) genes, as predicted by MFannot, were aligned with sequences of *Escherichia coli*. *E. coli* sequences were obtained from http://rna.ucsc.edu/rnacenter/ribosome_images.html, and their predicted domains [82, 83] are shown as magenta and blue boxes below the sequences. Nucleotide identities are shown by black background with white nucleotides.

## Data Availability

All data needed to evaluate the conclusions in the paper are present in the paper and/or the Supplementary Information files. Raw sequencing reads have been previously deposited at NCBI Sequence Read Archive (SRP102236) under NCBI BioProject PRJNA379597 [4]. Final assemblies are available from Figshare at https://figshare.com/s/de8998f2e6cc71de6702. BLAST server for assemblies is accessible at http://evocellbio.com/SAGdb/zahonova_et_al/. Additional data related to this paper may be requested from the authors.
