# Supplementary material for: Single-cell genomics unveils a canonical origin of the diverse mitochondrial genomes of euglenozoans
Source: BMC Biol. 2021 May 17;19:103. doi: 10.1186/s12915-021-01035-y (PMC8130358; doi:10.1186/s12915-021-01035-y)
Supplement: Supplementary file 2 — Additional file 2: Fig. S1. BlobTools plots showing contamination of several sequenced SAGs. The bacterial contamination is shown as blue circles, while sequences with eukaryotic signal are in magenta. For comparison, BlobTools plots for SAGs EU17 and EU18 are also shown. Fig. S2. 18S rDNA phylogeny of eukaryotes. The Maximum Likelihood phylogenetic tree was estimated from an alignment containing 131 taxa and 1551 nucleotide positions under the GTR + Γ model with standard bootstrapping (BS) and ultrafast bootstrapping (UFB). Support values are shown if ≥ 50% and ≥ 75% for BS and UFB, respectively. Fully supported nodes are shown as black circles. Fig. S3. 18S rDNA phylogeny of euglenozoans. The Maximum Likelihood phylogenetic tree was estimated from an alignment containing 368 taxa and 1269 nucleotide positions under the GTR + Γ model with standard bootstrapping. Support values are shown if ≥ 50%. Fig. S4. Predicted domains in EU17/18 mtDNA-encoded ORFs. ORFs annotated by MFannot (https://megasun.bch.umontreal.ca/cgi-bin/dev_mfa/mfannotInterface.pl) were submitted to an InterProScan [76] search. Predicted domains are highlighted as explained in the graphical legend. Fig. S5. Concatenated mitochondrial phylogenetic analysis including atp6 from EU17/18. The alignment contained 37 taxa and 4348 amino acid positions, with EU17/18 missing 3.65% of data. The Maximum Likelihood tree was estimated under two models, LG + C20 + F + Γ (C20) and LG + F + I + G4 (LG; the best-fitting model as determined by IQ-TREE), with 1000 standard bootstraps (BS) and 1000 ultrafast bootstraps (UFB). The tree topology shown is from the C20 analysis. Support values for < 50% BS and < 75% UFB are denoted by a dash (-), whereas an asterisk (*) marks a topology that does not exist in a particular analysis. Fully supported nodes are shown as black circles. Fig. S6. Characterization of tRNAs encoded in mtDNA of EU17/18 and EU2. a-b) Sequences of trnK (a) and trnM (b) were aligned with mitochondrial [file 12915_2021_1035_MOESM2_ESM.pdf]

Single-cell genomics unveils a canonical origin of the diverse mitochondrial genomes of euglenozoans

Kristína Záhonová[1,2], Gordon Lax[3], Savar D. Sinha[4], Guy Leonard[5], Thomas A. Richards[5], Julius Lukeš[1,6,*], Jeremy G. Wideman[4,*]

[1] Institute of Parasitology, Biology Centre, Czech Academy of Sciences, České Budějovice (Budweis), Czech Republic

[2] Faculty of Science, Charles University, BIOCEV, Vestec, Czech Republic

[3] Department of Botany, University of British Columbia, Vancouver, Canada

[4] Center for Mechanisms of Evolution, Biodesign Institute, School of Life Sciences, Arizona State University, Tempe, USA

[5] Department of Zoology, University of Oxford, Oxford, UK

[6] Faculty of Sciences, University of South Bohemia, České Budějovice (Budweis), Czech Republic

[*] Corresponding authors: jeremy.wideman@asu.edu; jula@paru.cas.cz

Additional file 2: Fig. S1-S7

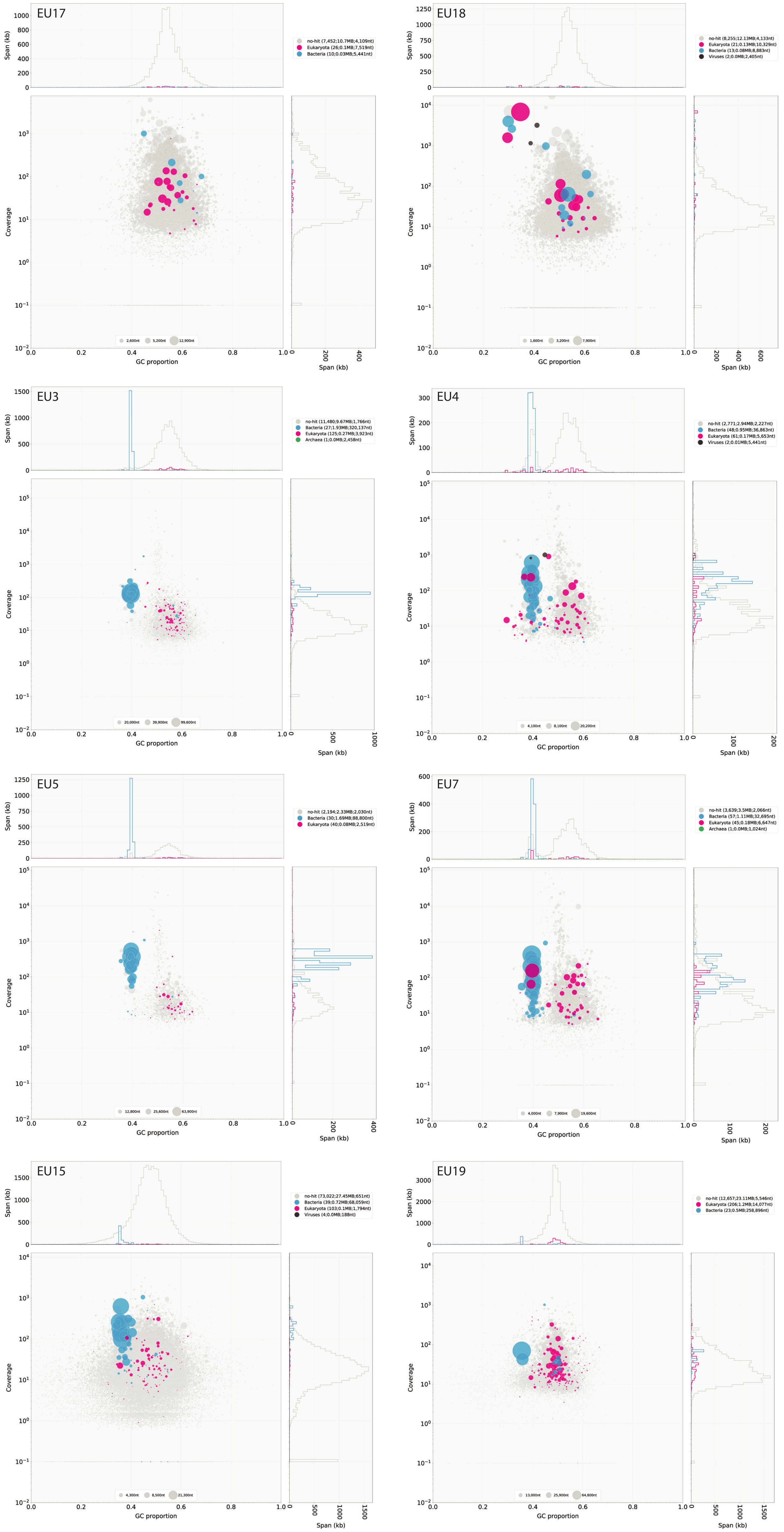

Fig S1. BlobTools plots showing contamination of several sequenced SAGs. The bacterial contamination is shown as blue circles, while sequences with eukaryotic signal are in magenta. For comparison, BlobTools plots for SAGs EU17 and EU18 are also shown.

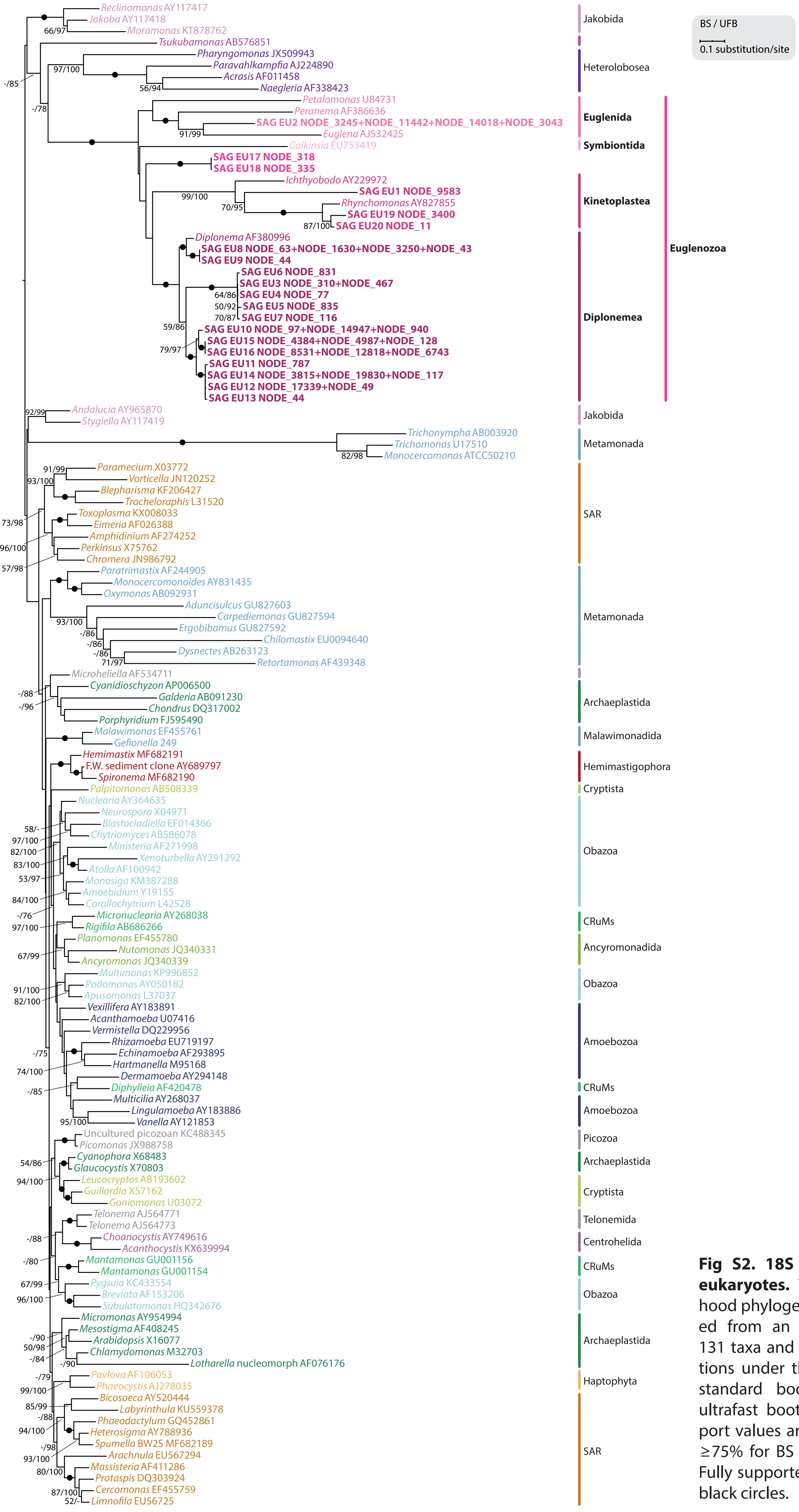

Fig S2. 18S rDNA phylogeny of eukaryotes. The Maximum Likelihood phylogenetic tree was estimated from an alignment containing 131 taxa and 1,551 nucleotide positions under the GTR+Γ model with standard bootstrapping (BS) and ultrafast bootstrapping (UFB). Support values are shown if ≥50% and ≥75% for BS and UFB, respectively. Fully supported nodes are shown as black circles.

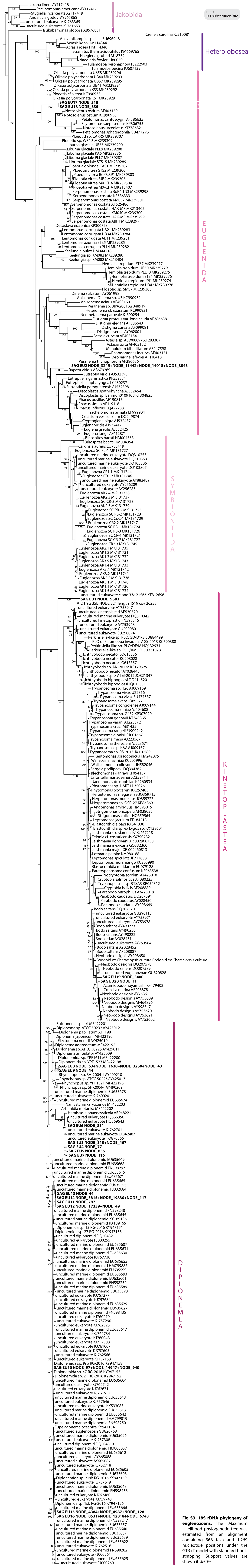

Fig S3. 18S rDNA phylogeny of euglenozoans. The Maximum Likelihood phylogenetic tree was estimated from an alignment containing 368 taxa and 1,269 nucleotide positions under the GTR+Γ model with standard boot-strapping. Support values are shown if ≥50%.

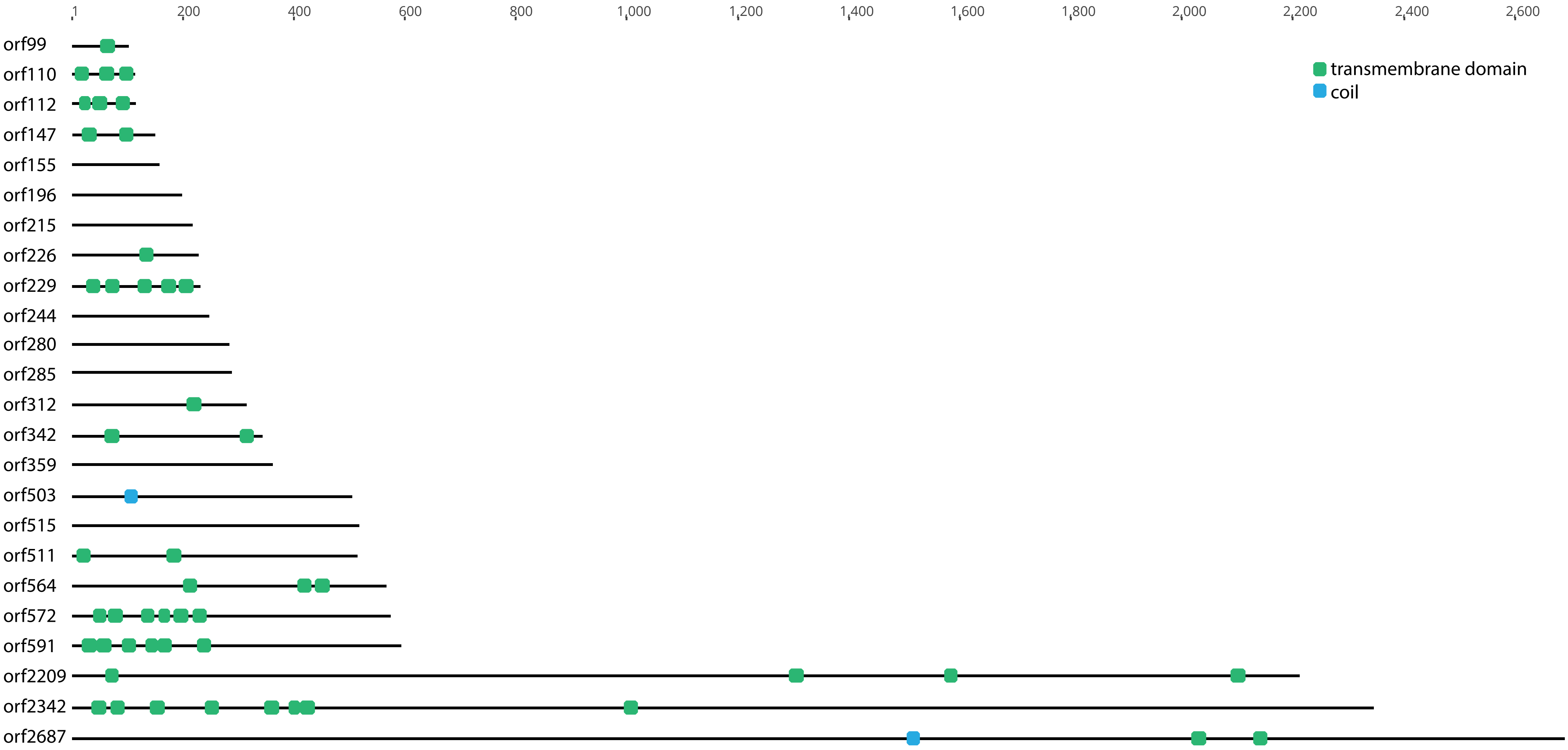

Fig S4. Predicted domains in EU17/18 mtDNA-encoded ORFs. ORFs annotated by MFannot (https://megasun.bch.umontreal.ca/cgi-bin/dev_mfa/mfannotInterface.pl) were submitted to anInterProScan [75] search. Predicted domains are highlighted as explained in the graphical legend.

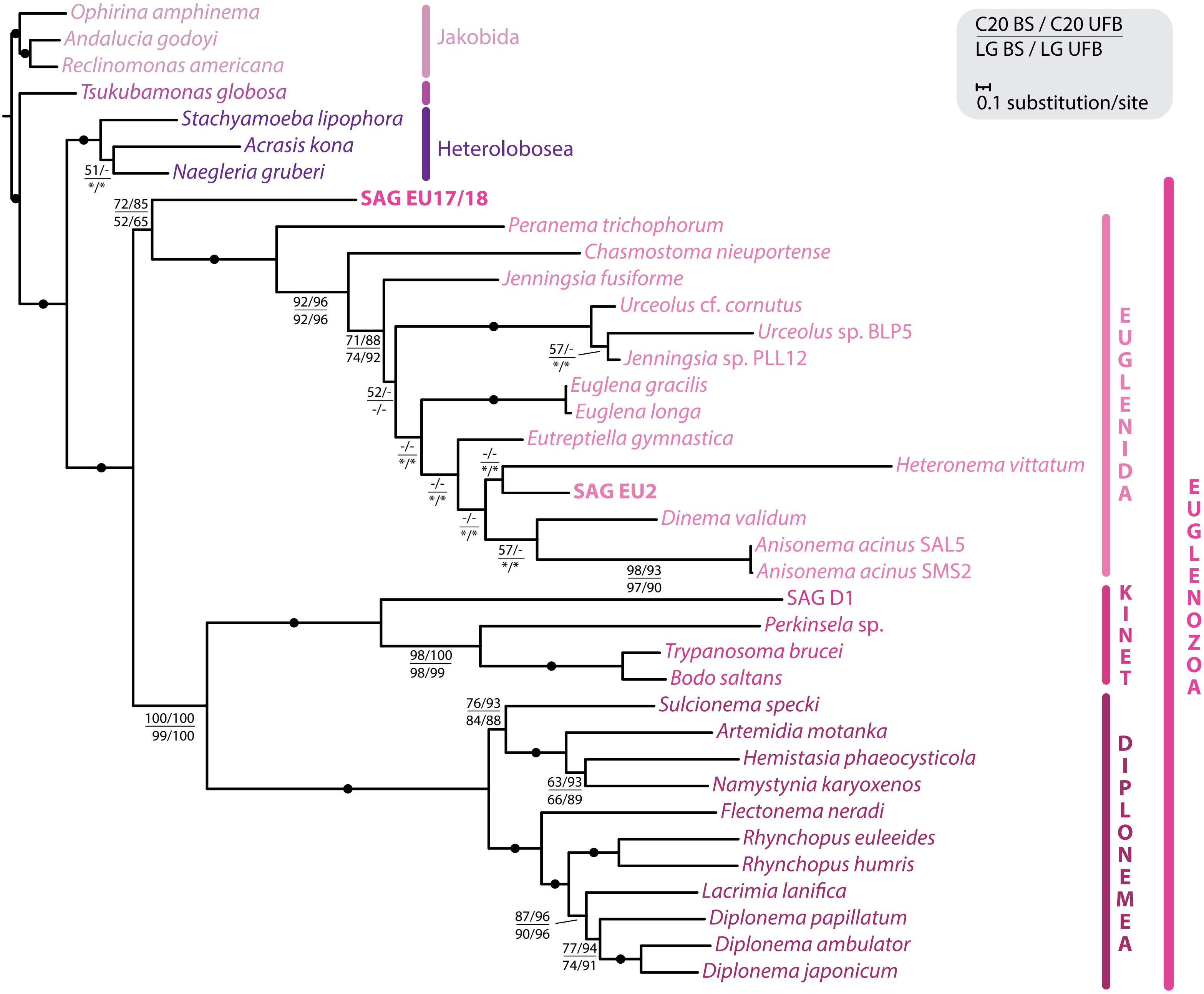

Fig. S5. Concatenated mitochondrial phylogenetic analysis including atp6 from EU17/18. The alignment contained 37 taxa and 4,348 amino acid positions, with EU17/18 missing 3.65% of data. The Maximum Likelihood tree was estimated under two models, LG+C20+F+Γ (C20) and LG+F+I+G4 (LG; the best-fitting model as determined by IQ-TREE), with 1,000 standard bootstraps (BS) and 1,000 ultrafast bootstraps (UFB). The tree topology shown is from the C20 analysis. Support values for ≤50% BS and ≤75% UFB are denoted by a dash (-), whereas an asterisk (*) marks a topology that does not exist in a particular analysis. Fully supported nodes are shown as black circles.

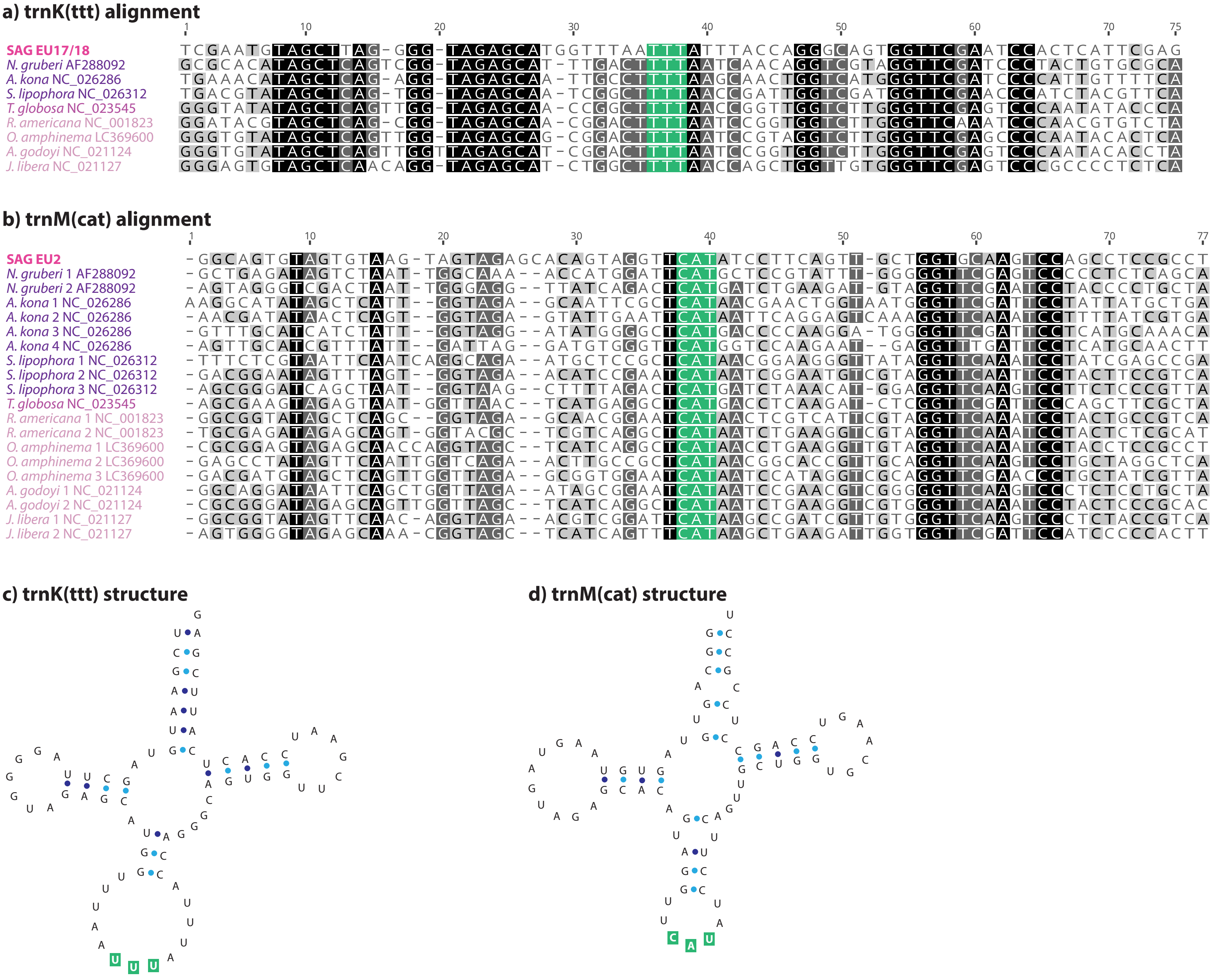

Fig S6. Characterization of tRNAs encoded in mtDNA of EU17/18 and EU2. a-b) Sequences of *trnK* (**a**) and *trnM* (**b**) were aligned with mitochondrially encoded tRNAs of other species of Discoba. Residue shading indicates sequence conservation. **c-d)** Secondary structures of *trnK* (**c**) and *trnM* (**d**) as predicted by tRNAScan-SE. Double and triple bonds are depicted as dark- and light-blue circles, respectively. Anticodons are highlighted with a green background. Since all other known euglenozoans import all tRNAs into mitochondria from the nucleus [47], we built tRNA alignments with homologues from the mtDNAs of other discobans to take into account different evolutionary pressures and mutational rates in nuclei and mitochondria [51]. The identity across nine *trnK* and 20 *trnM* sequences was 38.7% and 15.8%, respectively (**a-b**). Predicted secondary structures resembled other tRNAs supporting their functionality (**c-d**). While most eukaryotes have at least some tRNA mitochondrial-encoded, the long-standing paradigm was that euglenozoans and unrelated apicomplexans (which share with euglenozoans a range of unique features [77]) import all tRNAs from the cytosol [78]. This has significant consequences, since the bacterial-type translation system has to cope solely with the eukaryotic-type tRNAs [79].

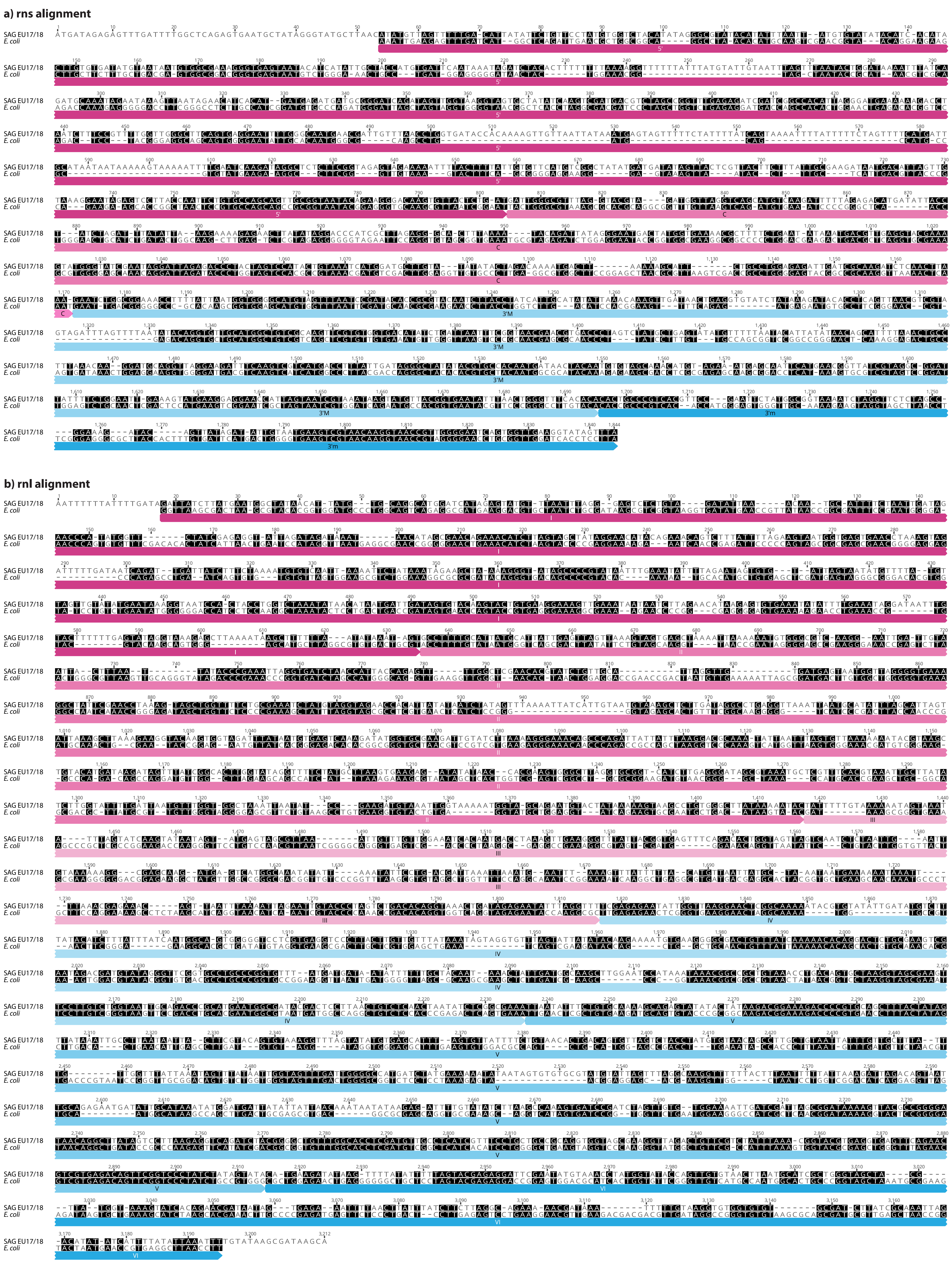

Fig. S7. Mitoribosomal RNAs of EU17/18. Sequences of *rns* (**a**) and *rnl* (**b**) genes, as predicted by MFannot, were aligned with sequences of *Escherichia coli*. *E. coli* sequences were obtained from http://rna.ucsc.edu/rnacenter/ribosome_images.html, and their predicted domains [80,81] are shown as magenta and blue boxes below the sequences. Nucleotide identities are shown by black background with white nucleotides.
